# Supplementary material for: Attitudes and opinions of medical practitioners, librarians, and LIS academics towards health science library services to support evidence-based medical practice in South Africa
Source: PLoS One. 2025 Aug 29;20(8):e0331507. doi: 10.1371/journal.pone.0331507 (PMC12396650; doi:10.1371/journal.pone.0331507)
Supplement: S1 File — (PDF) [file pone.0331507.s001.pdf]

Table S1: Questionnaire for Medical Practitioners.

QUESTIONNAIRE FOR MEDICAL PRACTITIONERS

The purpose of this questionnaire is to collect data for my DTech: Library and Information Studies research titled: Toward evidence based medical practice model for health science library services in public and private hospitals within a South African context.

Evidence based medical practice (EBMP) is the conscientious, explicit and judicious use of current best evidence/research in making decisions regarding the care of individual patients

PLEASE ANSWER THIS QUESTIONNAIRE BY PLACING A CROSS [X] IN THE CHECKBOX NEXT TO THE OPTION/S THAT APPLY IN EACH CASE. WHERE YOU SELECT 'OTHER', PLEASE EXPLAIN IN THE SPACE PROVIDED.

SECTION A: BIOGRAPHICAL DATA

1. Please indicate your gender

- [ ] Male
- [ ] Female

2. Please indicate age group

- [ ] 20-30
- [ ] 31-40
- [ ] 41-50
- [ ] 51-60
- [ ] 61-70
- [ ] 70+

3. Please indicate in which year you completed your last degree

[ ]

4. Please state your highest qualification and the discipline in which you attained this qualification

____

____

____

5. Please indicate your hospital where you are currently working

- [ ] Public
- [ ] Private
- [ ] Semi-private

6. For how many years have you been in medical practice?

- [ ] 0-5 yrs.
- [ ] 6-10 yrs.
- [ ] 11-15 yrs.
- [ ] 16-20 yrs.
- [ ] 21+ yrs.

7. Your specialization or discipline and job title?

____

____

8. On average, how many hours per week do you work?

- ☐ 1-20
- ☐ 21-30
- ☐ 31-40
- ☐ 41+

9. On average, how many patients do you see daily?

- ☐ 1-10
- ☐ 11-20
- ☐ 21-30
- ☐ 31-40
- ☐ 41+

SECTION B: ATTITUDES AND OPINIONS ON EBMP AND UNDERSTANDING OF MEDICAL PRACTICE GUIDELINES:

This section of the questionnaire inquires about personal attitudes toward, use of, and perceived benefits and limitations of EBMP.

For the following items, place a mark **X** in the appropriate box that indicates your response. In items referring to your "facility," consider the practice setting in which you do the majority of your clinical care.

	STATEMENT	AGREE	DISAGREE	NEUTRAL
10.	I am familiar with EBMP			
11.	Application of EBMP is necessary in my specialization or practice			
12.	EBMP is useful in my day-to-day practice			
13.	I need to increase the use of EBMP in my daily practice			
14.	I learned the foundations for EBMP as part of my academic preparation at medical school/university			
15.	EBMP improves the quality of patient care			
16.	I am familiar with the online medical search engines (e.g., MEDLINE, CINAHL)			

If you would like to add any information on the above questions, please explain

____

____

____

17. I can access relevant databases and the Internet at my facility

- ☐ Yes
- ☐ No
- ☐ Do not know

18. Do you think that evidence-based practice is necessary in medicine?

- [ ] Yes
- [ ] No

Please explain

____
____
____

19. What sources do you use to practice EBM?

- [ ] Print source
- [ ] Online and electronic sources
- [ ] Other____

20. If you use print sources, what sub source/s do you use?

- [ ] Books
- [ ] Journals
- [ ] Thesis/research reports
- [ ] Atlases
- [ ] Guidelines
- [ ] Others (please mention)

____
____

21. If you use online and electronic sources, what sub source/s do you use?

- [ ] Free web
- [ ] The Cochrane library
- [ ] PubMed
- [ ] Systematically reviewed literature
- [ ] Medline
- [ ] E-Journals
- [ ] Others (please mention)

____
____

22. Which website do you use in your practice of EBM?

- [ ] Medscape
- [ ] E-Medicine
- [ ] MD consult
- [ ] Others (please specify)

____
____

23. What are the reasons for your preferences for the use of print sources?

- [ ] Ease to use
- [ ] Easily available
- [ ] Currency
- [ ] Easy to carry
- [ ] Cost effectiveness
- [ ] Others (please mention)

____
____

24. What barriers/problems have you faced in EBMP? You may choose more than one answer

- [ ] Lack of personal time
- [ ] Patient overload
- [ ] EBM is difficult to understand
- [ ] Lack of interest
- [ ] Lack of library services
- [ ] Poor ability to critically appraise the literature

- [ ] Lack of information resources
- [ ] the absence of an effective computer system
- [ ] Lack of research skills
- [ ] Lack of understanding of statistical analysis
- [ ] Lack of EBM training courses
- [ ] Lack of resources and facilities
- [ ] Lack of collective support among my colleagues in my facility
- [ ] Inability to apply research findings to individual patients with unique characteristics
- [ ] Lack of generalizability of the literature findings to my patient population
- [ ] Other (please specify)

____
____
____

SECTION C: MEDICAL PRACTITIONER'S RESPONSE TOWARDS MEDICAL/HOSPITAL LIBRARY SERVICES:

25. Is there a library in the hospital in which you are practicing?

- [ ] Yes
- [ ] No

26. If you answered "YES" to question 25, please answer the following questions. If you answered "No" Please proceed to question 27. Explain your answer in each case.

26.1 Are you satisfied with the library services in your hospital?

- [ ] Yes
- [ ] No

26.2 If you are not satisfied with the library services in your hospital, what improvements would you like to see implemented?

____
____
____

26.3 Are you assisted by a librarian/s in term of your practice?

- [ ] Yes
- [ ] No

26.4 Are there librarians dedicated to your field of medicine/specialization?

- [ ] Yes
- [ ] No

26.5 What type of support or services do the librarians provide?

____
____
____

26.6 How often do you use the services of the librarians?

- [ ] Everyday
- [ ] Once a week
- [ ] Twice a week
- [ ] Once a month
- [ ] Twice a month
- [ ] Others (please specify)

____
____

26.7 Do you think that the librarians are adequately trained to assist you in your practice?

- [ ] Yes
- [ ] No

26.8 How do you think librarians should be trained to assist with EBMP?

____

____

26.9 Do you think that the librarians are suitably qualified to assist with EBMP?

☐ Yes
☐ No

26.10 Do you require the services of a librarian with expertise/knowledge of EBMP?

☐ Yes
☐ No

Please explain your answer ____

____

27. If you answered "NO" to question 25, please answer the following questions. Please explain your answer in each case

27.1. Do you think that library services should be established in your hospital?

☐ Yes
☐ No

Please explain your answer ____

____

27.2. If you do not use the services of a librarian, how do you access information for enhanced patient care?

____

____

28. Please indicate your response to each the questions in the table below

	STATEMENT<br>In each of the statements below, **circle the option** (**from the words in bold**) that apply to the hospital in which you practice	AGREE	DISAGREE	NEUTRAL
28.1	The services of librarians are **present** / **required** to support EBMP in hospitals			
28.2	Librarians **can / do** assist medical practitioners especially in complicated cases			
28.3	Librarians **can / do** assist medical practitioners with research/literature in cases where little is known about a disease or illness			
28.4	Librarians **can / do** assist medical practitioners with literature especially in the case of infectious diseases			
28.5	Librarians **can / do** assist medical practitioners to keep up to date with research/literature in their field			
28.6	Librarians **can / do** play a critical role in providing relevant information for individual cases to medical practitioners			
28.7	Librarians **can / do** save medical practitioners time by assisting them with their research			

29. Any comment positive or negative that you would like to make regarding library services in the hospitals

____

____

Thank you very much for your valuable opinions, patience and participating in the survey.

Table S2*:* **Questionnaire for Health Science Librarians.**

DUT DURBAN UNIVERSITY OF TECHNOLOGY

QUESTIONNAIRE FOR HEALTH LIBRARIANS

The purpose of this questionnaire is to collect data for my D. Tech: Library and Information Studies research titled: **Toward evidence based medical practice model for health science library services in public and private hospitals within a South African context.**

Evidence based medical practice (EBMP) is the conscientious, explicit and judicious use of current best evidence/research in making decisions regarding the care of individual patients

PLEASE ANSWER THIS QUESTIONNAIRE BY PLACING A CROSS [X] IN THE CHECKBOX NEXT TO THE OPTION/S THAT APPLY IN EACH CASE. WHERE YOU SELECT 'OTHER', PLEASE EXPLAIN IN THE SPACE PROVIDED.

SECTION A: BIOGRAPHIGAL DATA

1. Please indicate your gender

- [ ] Male
- [ ] Female

2. Please indicate your age group

- [ ] 20-30
- [ ] 31-40
- [ ] 41-50
- [ ] 51-60
- [ ] 61-70
- [ ] 70+

3. Please indicate your hospital where you are currently working

- [ ] Public
- [ ] Private
- [ ] Semi-private

4. How long have you been a librarian in hospital?

- [ ] 0-5 yrs.
- [ ] 6-10 yrs.
- [ ] 11-15 yrs.
- [ ] 16-20 yrs.
- [ ] 21+ yrs.

5. What is your job title?

____

____

6. What were the requirements of your present job in terms of your:

Qualification

____

Experience

____

Specialization
____
Training
____

7. Please describe your main job functions.
____
____

8. Does your work require you to have specialised knowledge of EBMP?

- [ ] Yes
- [ ] No

Please explain, ____
____

SECTION B: QUALIFICATION AND TRAINING

9. Please indicate in which year you completed your last degree

[ ]

10. Please state your highest qualification and the discipline in which you attained this qualification .
____
____

11. Where /from which University did you graduate?

- [ ] Zululand
- [ ] Limpopo
- [ ] Pretoria
- [ ] Western Cape
- [ ] Cape Town
- [ ] South Africa
- [ ] Fort Hare
- [ ] Walter Sisulu
- [ ] KwaZulu-Natal
- [ ] Durban University of Technology
- [ ] Other,

please pecify____
____

12. Did you have an option to specialise in supporting EBMP?

- [ ] Yes
- [ ] No

Please explain, ____
____

13. Did you do any in-service/practical work/work integrated learning at a hospital or medical facility as part of your qualification?

- [ ] Yes
- [ ] No

14. If you answered “YES” to 13 above, please answer the following questions if your answer “NO” please proceeds to question 15.

14.1 What was the nature of your in-service/practical work/work integrated?
____
____

14.2 Where did you do the above?
____
____

14.3 Who placed you /assisted you to get the above?

____
____

14.4 What was the duration of the above?

____
____

14.5 Did you benefit from the experience?

- [ ] Yes
- [ ] No

Please explain, ____
____

SECTION C: HEALTH SCIENCE LIBRARIAN'S RESPONSE TOWARD EBMP TRAINING

15. Do you think that your qualification prepared you adequately to support EBMP/become a health librarian?

- [ ] Yes
- [ ] No

Please explain, ____
____
____

16. Have you attended any course/s or training (other than your formal Library qualification) related to EBMP/health librarian?

- [ ] Yes
- [ ] No

Please provide detail,

____
____

17. If you answered "YES" to 16 above, please answer the following questions if you answer "NO" please proceed to question 18.1

17.1. What was the nature of the course/s or training you attended?

____
____

17.2. What was the name/title of the course/training?

____
____

17.3. Where did you attend the above?

____
____

17.4. What was the duration of the course/s or training?

____
____

17.5. What were the entrance requirements for the above?

____

17.6. How did the course/training benefit you for your current position?

____

17.7. What was the reason/s that you attended the above?

____

____

____

18. *If you answered “NO” to 16 above, please answer the following questions*

18.1. What was the reason/s that you did not attend any course/s or training related to EBMP?

____

____

18.2. Do you plan to attend any course/s or training related to EBMP in the near future? Please explain why

____

____

19. Do you work with specialist medical practitioners?

- [ ] Yes
- [ ] No

20. *If you answered “YES” to 19, please answer the following questions if you answer “NO” please proceed to question 21.1*

20.1. Which specialists do you work with?

____

____

20.2. How often do you work with them?

- [ ] Everyday
- [ ] Once a week
- [ ] Twice a week
- [ ] Once a month
- [ ] Twice a month

20.3. What service/s do you provide?

____

____

20.4. What is your involvement with EBMP?

____

____

____

21. *If you answered NO to 19, please answer the following question*

21.1. To whom do you provide library services?

____

____

21.2. Do you provide a general service to all medical practitioners in the hospital?

- [ ] Yes
- [ ] No

Please explain, ____

SECTION D: ATTITUDES AND OPINIONS ON RESEARCH

22. Does your job responsibility require expertise with EBMP resources (e.g., MEDLINE, EBM Reviews or the Cochrane collection)?

- ☐ Yes
- ☐ No

23. Do you have the requisite expertise to deal with these resources?

- ☐ Yes
- ☐ No

24. If you answered "YES" to question 22, where did you receive training?

____
____
____

25. If you answered "NO" to question 22, from where would you like to receive training in this regard?

____
____

26. Do you have adequate resources in your library to support EBMP?

- ☐ Yes
- ☐ No

If no, please specify,

____
____

27. What challenges or problems do you face in offering EBM services?

- ☐ Lack of organizational support
- ☐ Lack of trained staff
- ☐ Lack of time
- ☐ Lack of resources
- ☐ Other,

Please specify ____
____

28. Any other comments (positive or negative) that you would like to make regarding librarian's responsibilities, roles, qualification, training or job functions related to supporting EBMP.

____
____
____

29. Any comment (positive or negative) that you would like to make regarding library services for EBMP.

____
____

Thank you very much for your valuable opinions, patience and participating in the survey.

Table S3: Questionnaire for Academic Staff at the University.

DUT DURBAN UNIVERSITY OF TECHNOLOGY

Questionnaire for Academic Staff at the University

The purpose of this questionnaire is to collect data for my DTech: Library and Information Studies research titled: **Toward evidence based medical practice model for health science library services in public and private hospitals within a South African context.**

Evidence based medical practice (EBMP) is the conscientious, explicit and judicious use of current best evidence/research in making decisions regarding the care of individual patients

PLEASE ANSWER THIS QUESTIONNAIRE BY PLACING A CROSS [X] IN THE CHECKBOX NEXT TO THE OPTION/S THAT APPLY IN EACH CASE. WHERE YOU SELECT 'OTHER', PLEASE EXPLAIN IN THE SPACE PROVIDED.

SECTION A: BIOGRAPHICAL DATA

1. Please indicate your gender

- [ ] Male
- [ ] Female

2. Please indicate your age group

- [ ] 20-30
- [ ] 31-40
- [ ] 41-50
- [ ] 51-60
- [ ] 61-70
- [ ] 70+

3. Please indicate in which year you completed your last degree

____
____

4. Please state your highest qualification and the discipline in which you attained this qualification

____
____

5. Please state the name of your department and the university where you are currently working

____
____

6. How long have you been working in this department/programme?

- [ ] 0-5 yrs.
- [ ] 6-10 yrs.
- [ ] 11-15 yrs.

- [ ] 16-20 yrs.
- [ ] 21+ yrs.

7. What is your job title?

____

____

8. Where /from which University did you graduate?

- [ ] Zululand
- [ ] Limpopo
- [ ] Pretoria
- [ ] Western Cape
- [ ] Cape Town
- [ ] South Africa
- [ ] Fort Hare
- [ ] Walter Sisulu
- [ ] KwaZulu-Natal
- [ ] Durban University of Technology
- [ ] Other,

please specify ____

____

SECTION B: ATTITUDES AND OPINIONS ON EBMP

9. Have you heard about evidence based medicine (EBM) or evidence based medical practice (EBMP)?

- [ ] Yes
- [ ] No

10. Does you department offer any courses/training for librarians to support EBMP?

- [ ] Yes
- [ ] No

11. If you answered "yes" to question 10, please answer the following questions if your answer "NO" please proceeds to question 12.1

11.1 What is the nature of the course/s or training offered?

____

____

____

11.2 What is the name/title of the course/training?

____

____

11.3 What is the duration of the course/s or training?

____

____

11.4 What are the entrance requirements for the above?

____

____

11.5 What are students taught in the above?

____

____

11.6 Is there a big demand for the above from students? Please explain.

____

11.7 Is there a big demand for the above from medical practitioners/hospitals, etc? Please explain.

____

____

11.8 How many students are currently enrolled for the above?

11.9 How many students have completed the above?

11.10 Do you have staff members that are qualified or specifically trained to teach library support for EBMP?

12. If you answered “no” to question 10, please answer the following questions <u>Please substantiate your answer in EACH case:</u>

12.1 Does your department plan to offer the course/s or training in the near future?

12.2 Have students ever enquired about the above?

12.3 Have medical practitioners or the health sector ever enquired about the above?

- [ ] Yes
- [ ] No

12.4 Do you have any member/s from the medical fraternity on your Advisory Board? Have they ever requested that you offer library training to support EBMP?

13. Are students allowed to specialise in EBMP library services?

- [ ] Yes
- [ ] No

14. What theoretical training is provided for librarians working with EBMP?

15. What practical training is provided for librarians working with medical practitioners/ EBMP?

16. Is there a specialised module on EBMP support?

17. What is the duration of this module?

18. What aspects/sections are covered in this module please explain very briefly.

19. Are students required to do any in-service/practical work/work integrated learning at a hospital or medical facility library? Please specify.

- [ ] Yes
- [ ] No

19.1 Who places these students?

19.2. Which hospitals/medical facilities do they usually go to?

20. Any comment positive or negative that you would like to make regarding the preparedness, training or qualifications for health librarians supporting EBMP

Thank you for taking the time to complete this questionnaire

Table S4: Cronbach’s Alpha Results

Reliability Statistics

Cronbach's Alpha	Cronbach's Alpha Based on Standardized Items	N of Items
.709	.735	5

Item Statistics

	Mean	Std. Deviation	N
I am familiar with EBMP	1.2271	.60681	251
Application of EBMP is necessary in my specialization or practice	1.1036	.42571	251
EBMP is useful in my day-to-day practice	1.0996	.42195	251
I need to increase the use of EBMP in my daily practice	1.2311	.58855	251
EBMP improves the quality of patient care	1.1116	.45112	251

Reliability Statistics

Cronbach's Alpha	Cronbach's Alpha Based on Standardized Items	N of Items
.755	.759	3

Item Statistics

	Mean	Std. Deviation	N
are you satified with your library services at your hospital	1.8901	.31449	91
Are you assisted by a librarian/s in term of your practice?	1.8242	.38278	91
Are there librarians dedicated to your field of medicine/specialization?	1.8791	.32779	91

Reliability Statistics

Cronbach's Alpha	Cronbach's Alpha Based on Standardized Items	N of Items
.813	.905	7

Item Statistics

	Mean	Std. Deviation	N

The services of librarians are present / required to support EBMP in hospitals	3.3065	1.60302	248
Librarians can / do assist medical practitioners especially in complicated cases	1.2984	.78940	248
Librarians can / do assist medical practitioners with research/literature in cases where little is known about a disease or illness	1.1815	.65148	248
Librarians can / do assist medical practitioners with literature especially in the case of infectious diseases	1.2218	.69353	248
Librarians can / do assist medical practitioners to keep up to date with research/literature in their field	1.2056	.67492	248
Librarians can / do play a critical role in providing relevant information for individual cases to medical practitioners	1.2218	.69934	248
Librarians can / do save medical practitioners time by assisting them with their research	1.1815	.71092	248

Table S5: Chi-square Test Results

	Experience	Type of hospital	Working hours per week	Patients see every day	Familiarity with EBMP	Familiarity with online search engines	Need to increase the use of EBMP	EBMP is necessary for medical practice	EBMP is useful in day to day practice	Learn EBMP foundation in medical school	EBMP can improve the quality of patients care
Gender	<.001	.001	0.33	0.05	.077	.516	.006	.009	.053	.004	.566
Experience			.480	<.001		.686	.136	.136	.148	.009	.573
Age	<.001		<.001	<.001	0.16	.072	.342	.124	.618	.029	.005
Type of hospitals	<.001		.898	<.001	.014	.076	.008	.205	.211	.008	.999
Familiar with EBMP	.003	.014	.678	.045		.001	.006	<.001	<.001	.001	<.001
Application of EBMP is necessary in practice	.136	.205	.409	.187	<.001	.753	<.001		<.001	.218	<.001
Access of relevant databases and Internet	<.001	<.001	.789	<.001	0.107	.002	.643	.475	.770	.315	.567
Sources used to practice EBMP	.746	.005	.153	.230	.484	.028	.501	.696	.637	.143	<.001
Requirement of a librarian with expertise in EBMP	.742	.205	.600	.633	0.05	.016	.008	.008	<.001	.042	.005

The p-values for Experience by gender, age, and type of hospitals are <.001 and by familiarity with EBMP, access to relevant databases and the Internet, and learn EBMP foundation in medical school are .003, <.001, and .004, respectively. The *p*-value for gender by type of hospital needs to increase the use of EBMP. EBMP is necessary for medical practice, and learn EBMP foundation in medical school is .001, .006, .009, and .004. The *p*-values for age by experience, working hours per week, and patients seen per day are <.001, and EBMP can improve the quality of patient care at .005. The p-values for type of hospital by experience and patients see every day is <.001, by familiarity with EBMP is .014, and by need to increase the use of EBMP and learn EBMP foundation in medical school is .008. The *p*-values for familiarity with EBMP by experience are .003, by type of hospital, .014, by familiarity with online search engines and learn EBMP foundation in medical school, .001, and by EBMP is necessary for medical practice, EBMP is useful in day-to-day practice, and EBMP improves the quality of patient care is <.001. The *p*-values for application of EBMP is necessary in practice by familiarity with EBMP, need to increase the use of EBMP, EBMP useful in day to practice and EBMP can improve the quality of patient's care is <.001. The *p*-values for access of relevant databases and Internet by experience, type of hospital and patients see every day is <.001 by familiarity with online search engines is .002. The p-values for sources used to practice EBMP by type of hospital, familiarity with online search engines and EBMP can improve the quality of patient's care are .005, .028 and <.001. The *p*-values for requirement of a librarian with expertise in EBMP by familiarity with online search engines, need to increase the use of EBMP, EBMP is necessary for medical practice, EBMP is useful in day-to-day

practice, learn EBMP foundation in medical school and EBMP improve the quality of patient's care are .016, .008, .008. <.001, .042 and .005. The results of are discussed in more detail in chapter 4 with the Cross-tabulation tables.

Table S6. Demographic characteristics of medical practitioners.

Characteristics	Description	Number (%) (n=251)
Gender	Female	86 (34.3)
	Male	165 (65.7)
Age		
	20-30 years	24 (9.6)
	31-40 years	79 (31.5)
	41-50 years	74 (29.5)
	51-60 years	51 (20.3)
	61-70 years	12 (4.8)
	70+	11 (4.4)
Years of experience		
	0-5 years	39 (15.5)
	6-10 years	47 (18.7)
	11-15 years	78 (31.1)
	16-20 years	37 (14.7)
	21+years	50 (19.9)
Type of hospital where participants are currently working		
	Public	139 (55.3)
	Private	111 (44.2)
	Both	1 (0.4)

Table S7. Gender * Age Cross tabulation.

Gender of Participants			Please indicate your age group						Total
			20-30	31-40	41-50	51-60	61-70	70+	
	Male	Count	7	46	52	39	10	11	165
		% within gender	4.2%	27.9%	31.5%	23.6%	6.1%	6.7%	100.0%
	female	Count	17	33	22	12	2	0	86
		% within gender	19.8%	38.4%	25.6%	14.0%	2.3%	0.0%	100.0%
Total			24	79	74	51	12	11	251
			9.6%	31.5%	29.5%	20.3%	4.8%	4.4%	100.0%

Table S8. Gender vs Years of experience Cross tabulation.

Gender of Participants			Years of experience in medical practice					Total
			0-5 years	6-10 years	11-15 years	16-20 years	21+ years	
	Male	Count	13	29	56	23	44	165
		% within gender	7.9%	17.6%	33.9%	13.9%	26.7	100.0 %
	female	Count	26	18	22	14	6	86
		% within gender	30.2%	20.9%	25.6%	16.3%	7.0%	100.0 %
Total		Count	39	47	78	37	50	251

	% within gender	15.5%	18.7%	31.1%	14.7%	19.9%	100.0 %
(χ^2= 31.00, df = 4, P-value =< 001)							

Table S9. Gender * Type of hospital Cross tabulation. (χ2= 14.99, df = 2, P-value = .001 < 0.05)

Gender of Participants			Hospital where participants are currently working			Total
			Public	Private	both	
	Male	Count	77	87	1	165
		% within gender	46.7%	52.7%	.6%	100.0%
	female	Count	62	24	0	86
		% within gender	72.1%	27.9%	0.0%	100.0%
Total		Count	139	111	1	251
		% within gender	55.4%	44.2%	.4%	100.0%

Table S10. Year of completion of medical practitioners' last degree.

The year of completion of the last degree		Frequency	Percent	
	1980-1985	19	7.6%	
	1986-1990	11	4.4%	
	1991-1995	29	11.6%	
	1996-2000	32	12.7%	
	2001-2005	50	19.9%	
	2006-2010	71	28.3%	
	2011-2015	36	14.3%	
	Total	248	98.8%	
Non-participants		3	1.2%	
Total		251	100.0%	

n=248/251

Table S11. Medical practitioners' specialisation or job title.

		Frequency	Percent
	General surgery, specialist	54	21.5
	Family medicine	39	15.5
	G Physician	30	12.0
	Paediatrics	25	10.0
	Medical officer	21	8.4
	Gynaecologist	16	6.4
	Orthopaedic	11	4.4
	ENT	10	4.0
	Emergency medicine,	8	3.2
	Urologist	4	1.6
	Psychiatrist	4	1.6
	Osteoporosis	3	1.2
	Cardiologist	3	1.2
	Plastic surgeon	3	1.2
	Neurosurgeon	2	0.8
	Dentist	1	0.4

	Speech therapist	1	0.4
	Obstetrics	1	0.4
	Total	236	94.0
	No response	15	6.0
Total sample population		251	100.0

Table S12. Medical practitioners' specialisation or job title * Years of experience Cross tabulation.

Participant's specialisation or job title		Years of experience in medical practice 0-5 years	6-10 years	11-15 years	16-20 years	21+ years	Total
General Surgery, Specialist	Count	4	14	19	6	11	54
	% within specialisation and job title	7.4%	25.9%	35.2%	11.1%	20.4%	100.0%
Emergency medicine		5	1	1	1	0	8
		62.5%	12.5%	12.5%	12.5%	0.0%	100.0%
Family medicine		7	11	13	5	3	39
		17.9%	28.2%	33.3%	12.8%	7.7%	100.0%
Medical officer		8	5	3	2	3	21
		38.1%	23.8%	14.3%	9.5%	14.3%	100.0%
G Physician		4	5	11	5	5	30
		13.3%	16.7%	36.7%	16.7%	16.7%	100.0%
Paediatrics		1	3	7	6	8	25
		4.0%	12.0%	28.0%	24.0%	32.0%	100.0%
Osteoporosis		2	0	0	0	1	3
		66.7%	0.0%	0.0%	0.0%	33.3%	100.0%
Neurosurgeon		0	1	0	1	0	2
		0.0%	50.0%	0.0%	50.0%	0.0%	100.0%
Gynaecologist		1	2	3	3	7	16
		6.3%	12.5%	18.8%	18.8%	43.8%	100.0%
Cardiologist		0	0	3	0	0	3
		0.0%	0.0%	100.0%	0.0%	0.0%	100.0%
Orthopaedic		0	1	5	0	5	11
		0.0%	9.1%	45.5%	0.0%	45.5%	100.0%
ENT		0	0	5	5	0	10
		0.0%	0.0%	50.0%	50.0%	0.0%	100.0%
Urologist		0	1	3	0	0	4
		0.0%	25.0%	75.0%	0.0%	0.0%	100.0%
Psychiatrist		0	0	0	1	3	4
		0.0%	0.0%	0.0%	25.0%	75.0%	100.0%
Dentist		1	0	0	0	0	1
		100.0%	0.0%	0.0%	0.0%	0.0%	100.0%
Plastic surgeon		1	0	0	0	2	3
		33.3%	0.0%	0.0%	0.0%	66.7%	100.0%
Speech therapist		0	0	1	0	0	1
		0.0%	0.0%	100.0%	0.0%	0.0%	100.0%
Obstetrics		0	0	0	0	1	1
		0.0%	0.0%	0.0%	0.0%	100%	100.0%
Total		34	44	74	35	49	236

		14.4%	18.6%	31.4%	14.8%	20.8%	100.0%

Table S13. Medical practitioners' specialization or job title * Gender Cross tabulation.

Participant's specialisation or job title			Please indicate your gender		Total
			Male	Female	
	General Surgery, Specialist	Count	44	10	54
		% within specialisation or job title	81.5%	18.5%	100.0%
	Emergency medicine		5	3	8
			62.5%	37.5%	100.0%
	Family medicine		20	19	39
			51.3%	48.7%	100.0%
	Medical officer		8	13	21
			38.1%	61.9%	100.0%
	G Physician		18	12	30
			60.0%	40.0%	100.0%
	Paediatrics		17	8	25
			68.0%	32.0%	100.0%
	Osteoporosis		2	1	3
			66.7%	33.3%	100.0%
	Neurosurgeon		1	1	2
			50.0%	50.0%	100.0%
	Gynaecologists		11	5	16
			68.8%	31.3%	100.0%
	Cardiologist		3	0	3
			100.0%	0.0%	100.0%
	Orthopaedic		9	2	11
			81.8%	18.2%	100.0%
	ENT		9	1	10
			90.0%	10.0%	100.0%
	Urologist		4	0	4
			100.0%	0.0%	100.0%
	Psychiatrist		4	0	4
			100.0%	0.0%	100.0%
	Dentist		0	1	1
			0.0%	100.0%	100.0%
	Plastic surgeon		2	1	3
			66.7%	33.3%	100.0%
	Speech therapist		0	1	1
			0.0%	100.0%	100.0%
	Obstetrics		1	0	1
			100.0%	0.0%	100.0%
Total			158	78	236
			66.9%	33.1%	100.0%

Table S14. Average hours of weekly practice.

Working Hours per week	Frequency	Percent	Cumulative Percent
1-20	17	6.8	6.8
21-30	10	4.0	10.8
31-40	71	28.3	39.0
41+	153	61.0	100
Total	251	100	

Table S15. Average hours of weekly practice * Gender Cross tabulation.

On average, how many hours per week do you work?		Please indicate your gender		Total
		Male	female	
1-20	Count	9	8	17
	% average hours of weekly practice	52.9%	47.1%	100.0%
21-30		5	5	10
		50.0%	50.0%	100.0%
31-40		45	26	71
		63.4%	36.6%	100.0%
41+		106	47	153
		69.3%	30.7%	100.0%
Total		165	86	251
		65.7%	34.3%	100.0%

(χ^2= 3.36, df = 3, P-value = 0.33 > 0.05)

Table S16. Average number of patients daily examined.

Patients examined daily	Frequency	Percent	Cumulative percent
1-10	23	9.2	9.2
11-20	68	27.1	36.3
21-30	69	27.5	63.7
31-40	38	15.1	78.9
40+	53	21.1	100.0
Total	251	100.0	

Table S17. Average number of patients daily examined * Gender Cross tabulation.

On average, patient participants examine daily		Please indicate your gender		Total
		Male	female	
1-10	Count	17	6	23
	On average, what % patients do medical practitioners examine daily?	73.9%	26.1%	100.0%
11-20		49	19	68
		72.1%	27.9%	100.0%
21-30		47	22	69
		68.1%	31.9%	100.0%
31-40		17	21	38
		44.7%	55.3%	100.0%

40+		35	18	53
		66.0%	34.0%	100.0%
Total		165	86	251
		65.7%	34.3%	100.0%

(χ^2= 9.50, df = 4, P-value = 0.01)

Table S18. Average hours of weekly practice * Average number of patients daily examined Cross tabulation.

On average, how many hours per week do you work?			On average, how many patients do you examine daily?					Total
			1-10	11-20	21-30	31-40	40+	
	1-20	Count	2	7	7	0	1	17
		% of Total	.8%	2.8%	2.8%	0.0%	.4%	6.8%
	21-30	Count	1	0	3	3	3	10
		% of Total	.4%	0.0%	1.2%	1.2%	1.2%	4.0%
	31-40	Count	6	22	12	15	16	71
		% of Total	2.4%	8.8%	4.8%	6.0%	6.4%	28.3%
	41+	Count	14	39	47	20	33	153
		% of Total	5.6%	15.5%	18.7%	8.0%	13.1%	61.0%
Total		Count	23	68	69	38	53	251
		% of Total	9.2%	27.1%	27.5%	15.1%	21.1%	100.0%

(χ^2= 17.79, df = 12, P-value = 0.12 > 0.05)

Table S19. Age * Familiarity with EBMP Cross tabulation.(χ2= 14.16, df = 10, P-value = 0.16 > 0.05)

Participant's Age group			I am familiar with EBMP			Total
			Agree	disagree	neutral	
	20-30 Count		21	1	2	24
	% of Total		87.5%	4.2%	8.3%	100.0%
	31-40		61	6	12	79
			77.2%	7.6%	15.2%	100.0%
	41-50		69	2	3	74
			93.2%	2.7%	4.1%	100.0%
	51-60		45	0	6	51
			88.2%	0.0%	11.8%	100.0%
	61-70		11	0	1	12
			91.7%	0.0%	8.3%	100.0%
	70+		11	0	0	11
			100.0%	0.0%	0.0%	100.0%
Total			218	9	24	251
			86.9%	3.6%	9.6%	100.0%

Table S20. Years experience in medical practice * Familiarity with EBMP Cross tabulation.

Years of experience medical practice		I am familiar with EBMP			Total
		Agree	disagree	neutral	
0-5 years	Count	31	2	6	39
	% of Total	79.5%	5.1%	15.4%	100.0%
6-10 years		34	6	7	47
		72.3%	12.8%	14.9%	100.0%
11-15 years		72	1	5	78
		92.3%	1.3%	6.4%	100.0%
16-20 years		36	0	1	37
		97.3%	0.0%	2.7%	100.0%

21+ years	45<br>90.0%	0<br>0.0%	5<br>10.0%	50<br>100.0%
Total	218<br>86.9%	9<br>3.6%	24<br>9.6%	251<br>100.0%

(χ^2= 23.17, df = 8, P-value = 0.003 < 0.05)

Table S21. Type of hospital where medical practitioners work * Familiarity with EBMP Cross tabulation. (χ^2= 12.43, df = 4, P-value = .014 < 0.05)

Type of hospital where participants are currently working			I am familiar with EBMP			Total
			Agree	Disagree	Neutral	
Public	Count		112	9	18	139
	% of Total		80.6%	6.5%	12.9%	100.0%
Private			105	0	6	111
			94.6%	0.0%	5.4%	100.0%
both			1	0	0	1
			100.0%	0.0%	0.0%	100.0%
Total			218	9	24	251
			86.9%	3.6%	9.6%	100.0%

Table S22. Familiarity with EBMP * Recognition that EBMP is necessary for specialisation Cross tabulation. ($\chi2$= 26.77, df = 2, P-value = <0.001)

I am familiar with EBMP		Application of EBMP is necessary for specialisation or practice			Total
		Agree	Disagree	Neutral	
Agree	Count	212	1	5	218
	% of Total	84.5%	0.4%	2.0%	86.9%
disagree	Count	7	0	2	9
	% of Total	2.8%	0.0%	0.8%	3.6%
neutral	Count	17	3	4	24
	% of Total	6.8%	1.2%	1.6%	9.6%
Total	Count	236	4	11	251
	% of Total	94.0%	1.6%	4.4%	100.0%

Table S23. Familiarity with EBMP * Recognition that EBMP is useful in day-to-day practice Cross tabulation. ($\chi2$= 78.36, df = 4, P-value = < 0.001)

I am familiar with EBMP		EBMP is useful in day-to-day practice			Total
		Agree	Disagree	Neutral	
Agree	Count	216	1	1	218
	% of Total	86.1%	0.4%	0.4%	86.9%
disagree	Count	4	1	4	9
	% of Total	1.6%	0.4%	1.6%	3.6%
neutral	Count	17	1	6	24
	% of Total	6.8%	0.4%	2.4%	9.6%
Total	Count	237	3	11	251
	% of Total	94.4%	1.2%	4.4%	100.0%

Table S24. Recognition that EBMP is necessary for specialisation * Recognition that EBMP is useful in day-to-day practice Cross tabulation.($\chi2$= 175, df = 4, P-value = < .001)

Application of EBMP is necessary for specialisation or practice		EBMP is useful in my day-to-day practice			Total
		Agree	Disagree	Neutral	
Agree	Count	232	1	3	236
	% of Total	92.4%	0.4%	1.2%	94.0%
Disagree	Count	0	2	2	4
	% of Total	0.0%	0.8%	0.8%	1.6%
Neutral	Count	5	0	6	11

	% of Total	2.0%	0.0%	2.4%	4.4%
Total	Count	237	3	11	251
	% of Total	94.4%	1.2%	4.4%	100.0%

Table S25. Familiarity with EBMP * EBMP improves quality of patient care Cross tabulation.(χ2= 23.80, df = 4, P-value =<.001)

I am familiar with EBMP		EBMP improves the quality of patient care			Total
		Agree	Disagree	Neutral	
Agree	Count	210	2	6	218
	% of Total	83.7%	0.8%	2.4%	86.9%
disagree	Count	6	0	3	9
	% of Total	2.4%	0.0%	1.2%	3.6%
neutral	Count	20	0	4	24
	% of Total	8.0%	0.0%	1.6%	9.6%
Total	Count	236	2	13	251
	% of Total	94.0%	0.8%	5.2%	100.0%

Table S26. Familiarity with EBMP * Intention to increase EBMP in daily practice Cross tabulation.(χ2= 14.26, df = 4, P-value = .006 < 0.05)

I am familiar with EBMP		I need to increase the use of EBMP in my daily practice			Total
		Agree	Disagree	Neutral	
Agree	Count	190	15	13	218
	% of Total	75.7%	6.0%	5.2%	86.9%
disagree	Count	6	0	3	9
	% of Total	2.4%	0.0%	1.2%	3.6%
neutral	Count	18	1	5	24
	% of Total	7.2%	0.4%	2.0%	9.6%
Total	Count	214	16	21	251
	% of Total	85.3%	6.4%	8.4%	100.0%

Table S27. Familiarity with EBMP * Learned foundations for EBMP at medical school/university Cross tabulation.(χ2= 19.76, df = 4, P-value = .001 < 0.05)

I am familiar with EBMP		Learned the foundations for EBMP as part academic preparation at medical school/university			Total
		Agree	Disagree	Neutral	
Agree	Count	143	44	31	218
	% of Total	57.0%	17.5%	12.4%	86.9%
disagree	Count	1	3	5	9
	% of Total	0.4%	1.2%	2.0%	3.6%
neutral	Count	11	10	3	24
	% of Total	4.4%	4.0%	1.2%	9.6%
Total	Count	155	57	39	251
	% of Total	61.8%	22.7%	15.5%	100.0%

Table S28. Familiarity with EBMP * Familiarity with online medical search engines (e.g., MEDLINE, CINAHL) Cross tabulation. (χ2= 17.80, df = 4, P-value = .001 < 0.05)

I am familiar with EBMP		I am familiar with the online medical search engines (e.g., MEDLINE, CINAHL)			Total
		Agree	Disagree	Neutral	
Agree	Count	178	19	21	218

	% of Total	70.9%	7.6%	8.4%	86.9%
disagree	Count	5	3	1	9
	% of Total	2.0%	1.2%	0.4%	3.6%
neutral	Count	14	2	8	24
	% of Total	5.6%	0.8%	3.2%	9.6%
Total	Count	197	24	30	251
	% of Total	78.5%	9.6%	12.0%	100.0%

Table S29. Access of relevant databases and the Internet at work place.

Response		Frequency	Percent
	Yes	107	42.6
	No	131	52.2
	Total	238	94.8
	No response	13	5.2
Total sample population		251	100.0

n=238/251

Table S30. Hospital where participants are currently working * Access of relevant databases and the Internet at workplace Cross tabulation.

Type of hospital where participants are currently working	Access of relevant databases and the Internet at work place		Total
	Yes	No	
Public	42	87	129
	32.6%	67.4%	100.0%
Private	65	43	108
	60.2%	39.8%	100.0%
both	0	1	1
	0.0%	100.0%	100.0%
Total	107	131	238
	45.0%	55.0%	100.0%

(χ^2= 18.95, df = 2, P-value = < .001)

Table S31. Familiarity with EBMP * Access to relevant databases and the Internet at workplace cross tabulation.

Familiar with EBMP	Access of relevant databases and the Internet at work place		Total
	Yes	No	
Agree	95	112	207
	45.9%	54.1%	100.0%
disagree	1	8	9
	11.1%	88.9%	100.0%
neutral	11	11	22
	50.0%	50.0%	100.0%
Total	107	131	238
	45.0%	55.0%	100.0%

n=238/251

(χ^2= 4.46, df = 2, P-value = 0.107 >0.05)

Table S32. Sources used by medical practitioners to practice EBM.

	Frequency	Percent

	Print source	16	6.4
	Online and electronic sources	33	13.1
	Both	196	78.1
	Other	4	1.6
	Total	249	99.2
	No response	2	0.8
Total		251	100.0

Table S33. Type of hospital where medical practitioners work * Sources used by medical practitioners to practice EBM. Cross tabulation.

Type of hospital where participants are currently working		What sources do you use to practice EBM?				Total
		Print source	Online and electronic sources	Both	Other	
	Public	12<br>8.7%	17<br>12.3%	107<br>77.5%	2<br>1.4%	138<br>100.0%
	Private	3<br>2.7%	16<br>14.5%	89<br>80.9%	2<br>1.8%	110<br>100.0%
	Both	1<br>100.0%	0<br>0.0%	0<br>0.0%	0<br>0.0%	1<br>100.0%
Total		16<br>6.4%	33<br>13.3%	196<br>78.7%	4<br>1.6%	249<br>100.0%

(χ^2= 18.38, df = 6, P-value = 0.005 < 0.05)

Table S34. Medical practitioners' suggested improvements to library services. n=79/83

		Frequency	Percent
Valid	Electronic resources, up-to-date journals and books, access to the useful database.	31	37.3
	More computers and books and expert librarians.	38	45.7
	Internet	6	7.2
	Extended hours of library especially after hours.	4	4.8
	No response	4	4.8
Total		83	100.0

Table S35. Age group * Medical practitioners' suggested improvements to library services Cross tabulation.

Age group		If you are not satisfied with the library services in your hospital, what improvements would you like to see implemented?				Total
		Electronic resources, up-to-date journals and books, access to useful database	More computers, books and expert librarians	Internet	Extended hours of library especially after hours	
20-30	Count	7	1	0	0	8
	% within age group	87.5%	12.5%	0.0%	0.0%	100.0%
31-40		20<br>54.1%	15<br>40.5%	1<br>2.7%	1<br>2.7%	37<br>100.0%
41-50		4<br>26.7%	9<br>60.0%	0<br>0.0%	2<br>13.3%	15<br>100.0%
51-60		0<br>0.0%	10<br>71.4%	3<br>21.4%	1<br>7.1%	14<br>100.0%

61-70	0	1	0	0	1
	0.0%	100.0%	0.0%	0.0%	100.0%
70+	0	2	2	0	4
	0.0%	50.0%	50.0%	0.0%	100.0%
Total	31	38	6	4	79
	39.2%	48.1%	7.6%	5.1%	100.0%

Table S36. Age group * Medical practitioners' suggested improvements to library services Cross tabulation.

Age group		If you are not satisfied with the library services in your hospital, what improvements would you like to see implemented?				Total
		electronic resources, up-to- date journals and books, access to the useful database	more computers and books and expert librarians	Internet	Extended hours of library especially after hours	
20-30	Count	7	1	0	0	8
	% of Total	8.9%	1.3%	0.0%	0.0%	10.1%
31-40		20	15	1	1	37
		25.3%	19.0%	1.3%	1.3%	46.8%
41-50		4	9	0	2	15
		5.1%	11.4%	0.0%	2.5%	19.0%
51-60		0	10	3	1	14
		0.0%	12.7%	3.8%	1.3%	17.7%
61-70		0	1	0	0	1
		0.0%	1.3%	0.0%	0.0%	1.3%
70+		0	2	2	0	4
		0.0%	2.5%	2.5%	0.0%	5.1%
Total		31	38	6	4	79
		39.2%	48.1%	7.6%	5.1%	100.0%

Table S37. Support or services provided by librarians. n=67/93

		Frequency	Percent
Valid	nil, not helpful	44	47.3
	do not know	1	1.1
	basic	5	5.4
	book search	14	15
	data access	3	3.2
	Total	67	72
No	response	26	28
Total sample population		93	100.0

Table S38. Age group * Support or services provided by librarians Cross tabulation.

Please indicate your age group		What type of support or services do the librarians provide?					Total
		nil, not helpful	do not know	basic	book search	data access	
20-30	Count	6	0	0	0	0	6
	% of Total	9.0%	0.0%	0.0%	0.0%	0.0%	9.0%
31-40	Count	22	1	1	3	3	30
	% of Total	32.8%	1.5%	1.5%	4.5%	4.5%	44.8%
41-50	Count	10	0	1	4	0	15
	% of Total	14.9%	0.0%	1.5%	6.0%	0.0%	22.4%
51-60	Count	5	0	1	3	0	9
	% of Total	7.5%	0.0%	1.5%	4.5%	0.0%	13.4%

	61-70	Count	0	0	1	4	0	5
		% of Total	0.0%	0.0%	1.5%	6.0%	0.0%	7.5%
	70+	Count	1	0	1	0	0	2
		% of Total	1.5%	0.0%	1.5%	0.0%	0.0%	3.0%
Total		Count	44	1	5	14	3	67
		% of Total	65.7%	1.5%	7.5%	20.9%	4.5%	100.0%

Table S39. Support or services provided by librarians * Frequency of use of librarian services Cross tabulation.

Type of support or services does the librarians provide		How often do you use the services of librarians?							Total
		Twice a week	Once a month	Twice a month	Other	only once not readings available	stopped using it found not useful	never	
nil, not helpful	Count	1	6	8	10	8	11	0	44
	% of Total	1.5%	9.0%	11.9%	14.9%	11.9%	16.4%	0.0%	65.7%
do not know	Count	0	0	0	0	0	0	1	1
	% of Total	0.0%	0.0%	0.0%	0.0%	0.0%	0.0%	1.5%	1.5%
basic	Count	0	1	3	1	0	0	0	5
	% of Total	0.0%	1.5%	4.5%	1.5%	0.0%	0.0%	0.0%	7.5%
book search	Count	3	7	4	0	0	0	0	14
	% of Total	4.5%	10.4%	6.0%	0.0%	0.0%	0.0%	0.0%	20.9%
data access	Count	0	0	2	1	0	0	0	3
	% of Total	0.0%	0.0%	3.0%	1.5%	0.0%	0.0%	0.0%	4.5%
Total	Count	4	14	17	12	8	11	1	67
	% of Total	6.0%	20.9%	25.4%	17.9%	11.9%	16.4%	1.5%	100.0 %

Table S40. Age * Medical practitioners' perception of training of librarians to assist with EBMP Cross tabulation.

Please indicate your age group			Do you think that the librarians are adequately trained to assist you in your practice		Total
			Yes	No	
	20-30	Count	1	8	9
		% of Total	1.1%	8.6%	9.7%
	31-40	Count	7	32	39
		% of Total	7.5%	34.4%	41.9%
	41-50	Count	8	12	20
		% of Total	8.6%	12.9%	21.5%
	51-60	Count	5	11	16
		% of Total	5.4%	11.8%	17.2%
	61-70	Count	4	1	5

		% of Total	4.3%	1.1%	5.4%
	70+	Count	2	2	4
		% of Total	2.2%	2.2%	4.3%
Total		Count	27	66	93
		% of Total	29.0%	71.0%	100.0%

Table S41. Medical practitioner' opinions on training of librarians to assist with EBMP.

	Frequency	Percent
They need to be trained to assist in EndNote and electronic journal access.	4	4.3
They need to be made familiar with EBMP , critical appraisal literature reviews, and various services of information	22	23.7
They need to attend courses, workshops and acquire necessary qualifications for EBMP	37	39.8
They need to be trained in Database search	4	4.3
Do not know	2	2.1
Total	69	74.2
No response	24	25.8
Total population who have a library in their hospital	93	100

n=69/93

Table S42. Familiarity with EBMP * Medical practitioners' perception of training of librarians to assist with EBMP Cross tabulation.

I am familiar with EBMP		Do you think that the librarians are suitably qualified to assist with EBMP?			Total
		Yes	No	do not know	
Agree	Count	45	36	3	84
	% of Total	48.4%	38.7%	3.2%	90.3%
Disagree	Count	0	2	0	2
	% of Total	0.0%	2.2%	0.0%	2.2%
Neutral	Count	3	4	0	7
	% of Total	3.2%	4.3%	0.0%	7.5%
Total	Count	48	42	3	93
	% of Total	51.6%	45.2%	3.2%	100.0%

n=93

Table S43. Familiarity with EBMP * Medical practitioners' requirement of a librarian with expertise in EBMP Cross tabulation.

I am familiar with EBMP		Do you require the services of a librarian with expertise in EBMP?		Total
		Yes	No	
Agree	Count	77	5	82
	% of Total	82.8%	5.4%	88.2%

disagree	Count	2	1	3
	% of Total	2.2%	1.1%	3.2%
neutral	Count	7	1	8
	% of Total	7.5%	1.1%	8.6%
Total	Count	86	7	93
	% of Total	92.5%	7.5%	100.0%

n=93, (χ^2= 5.94, df = 2, P-value = 0.05 = 0.05)

Table S44. Medical practitioners' other information sources for patient care.

Participant's statement		Frequency	Percent
Valid	"By myself on my computer, Internet search, Google, online free journals, and Wikipedia"	97	61.4
	"Old notes of patients"	5	3.2
	"University libraries or medical school libraries"	12	7.6
	"Spend my own money on books"	7	4.4
	"Other hospital"	2	1.2
	"meetings, conference, discuss with colleagues"	6	3.8
No	response	29	18.4
Total		158	100.0

n=129/158 (who do not have a library in their hospital)

Table S45. Statements of medical practitioners about librarians.

STATEMENT<br>In each of the statements below, circle the option (from the words in bold) that apply to the hospital in which you practice.	Can assist agree (%)	Can assist disagree (%)	Can assist neutral (%)	Do assist agree (%)	Do assist disagree (%)	Do assist neutral (%)
1. Librarians can / do assist medical practitioners especially in complicated cases.	84.9	2.0	9.6	1.2	1.2	1.2
2. Librarians can / do assist medical practitioners with research/literature in cases where little is known about a disease or illness.	90.4	1.6	5.2	0.4	1.2	1.2
3. Librarians can / do assist medical practitioners with literature especially in the case of infectious diseases.	88	2.4	6.8	0.4	1.2	1.2
4. Librarians can / do assist medical practitioners to keep up-to-date with research/literature in their field.	88.8	2.4	6.0	0.4	1.2	1.2
5. Librarians can / do play a critical role in providing relevant information for individual cases to medical practitioners.	88.8	2.8	6.0	0.8	1.2	1.2
6. Librarians can / do save medical practitioners time by assisting them with their research,	91.6	0.8	4.0	0.8	1.2	1.6

Table S46: Gender, age, experience, and job title of health science librarians.

Characteristics	Description	Number (%) (n=5)
Gender	Female	4 (80)
	Male	1(20)
Age		
	20-30 years	0
	31-40 years	2 (40)
	41-50 years	3 (60)
	51-60 years	0
	61-70 years	0
	70+	0
Years of work experience		
	0-5 years	0
	6-10 years	3 (60)
	11-15 years	2 (40)
	16-20 years	0
	21+years	0
Job title of health science librarians		
	Assistant librarian	3 (60)
	Librarian	2 (40)

Table S47. Health science librarians' expertise with EBMP resources.

Question	Yes	No
Does your job responsibility require expertise with EBMP resources (e.g., MEDLINE, EBM Reviews or the Cochrane Library Collection)?	3	2
Do you have the requisite expertise to deal with these resources?	3	2

Table S48. Gender and age of academic staff.

Characteristics	Description	Number (%) (n=24)
Gender	Female	11 (45.8)
	Male	11 (45.8)
	No response	2 (8.4)
Age		
	20-30 years	1 (4.2)
	31-40 years	7 (29.2)
	41-50 years	4 (16.6)
	51-60 years	9 (37.5)
	61-70 years	3 (12.5)
	70+	0
